# Supplementary material for: The effect of a six-month programme of intradialytic cycling on survival and hospitalisations in people requiring haemodialysis: 5-year follow-up of the CYCLE-HD randomised controlled trial
Source: PLoS One. 2025 Sep 22;20(9):e0332389. doi: 10.1371/journal.pone.0332389 (PMC12453264; doi:10.1371/journal.pone.0332389)
Supplement: S1 Table — (DOCX) [file pone.0332389.s001.docx]

| **S1 Table.** ICD-10 codes used to define hospitalisations related to cardiovascular diagnoses |
| --- |
| **Hypertensive diseases:** 'I10' (Essential (primary) hypertension),'I11' (Hypertensive heart disease),'I12' (Hypertensive renal disease),'I13' (Hypertensive heart and renal disease),'I15' (Secondary hypertension),'I16' (Hypertensive crisis),'I1A' (Resistant hypertension). |
| **Ischaemic heart diseases:** 'I20' (Angina Pectoris),'I21' (Acute myocardial infarction),'I22' (Subsequent myocardial infarction),'I23' (Certain current complications following acute myocardial infarction),'I24' (Other acute ischaemic heart diseases),'I25' (Chronic ischaemic heart disease). |
| **Other forms of heart disease:** 'I34' (Nonrheumatic mitral valve disorders),'I35' (Nonrheumatic aortic valve disorders),'I36' (Nonrheumatic tricuspid valve disorders),'I37' (Pulmonary valve disorders), 'I42' (Cardiomyopathy),'I43' (Cardiomyopathy in diseases classified elsewhere),'I44' (Atrioventricular and left bundle-branch block),'I45' (Other conduction disorders),'I46' (Cardiac arrest),'I47' (Paroxysmal tachycardia),'I48' (Atrial fibrillation and flutter),'I49' (Other cardiac arrhythmias), 'I50' (Heart failure),'I51' (Complications and ill-defined descriptions of heart disease),'I52' (Other heart disorders in diseases classified elsewhere), ‘I5a’ (non-ischaemic myocardial injury (non traumatic). |
| **Cerebrovascular diseases:** 'I60' (Subarachnoid haemorrhage),'I61' (Intracerebral haemorrhage),'I62' (Other nontraumatic intracranial haemorrhage),'I63' (Cerebral infarction),'I65' (Occlusion and stenosis of precerebral arteries, not resulting in cerebral infarction),'I66' (Occlusion and stenosis of cerebral arteries, not resulting in cerebral infarction). |
| **Diseases of arteries, arterioles and capillaries:** 'I70' (Atherosclerosis) |
