# Supplementary material for: The effect of a six-month programme of intradialytic cycling on survival and hospitalisations in people requiring haemodialysis: 5-year follow-up of the CYCLE-HD randomised controlled trial
Source: PLoS One. 2025 Sep 22;20(9):e0332389. doi: 10.1371/journal.pone.0332389 (PMC12453264; doi:10.1371/journal.pone.0332389)
Supplement: Attachment — Submitted filename: PLOS One Response to Reviewers_01082025.docx [file pone.0332389.s003.docx]

1^st^ August 2025

Fan Zhang

Academic Editor

PLOS ONE

Dear Fan Zhang

We would like to thank the Reviewers for their careful evaluation of our initial submission, and providing us with specific comments and suggestions which we feel have significantly improved the overall quality of our manuscript. We now hope that it meets PLOS ONE’s publication criteria. We do not believe that the comments provided by Reviewer 2 relate to our manuscript. Therefore, we have not addressed these comments. Could you please provide us with some clarification around this please.

Please find our responses to all comments in the table below. We have resubmitted a tracked changes copy of the updated manuscript based on the reviewer comments, furthermore we have also submitted a clean copy. We believe that the manuscript now meets PLOS ONE’s style requirements (**Point 1**). For **Point 2**, we have removed the funding related text from the manuscript. For **Point 3**, (regarding data underlying the findings described in their manuscript to be freely available to other researchers), we have now uploaded the data underlying these findings to <https://doi.org/10.25392/leicester.data.29721590> so that they are freely available. **Point 4**, the title has been amended in the online submission form so that it is identical to the manuscript. For **Point 5**, we have removed the ethics statement from the end of the manuscript so that it now only appears in the methods section of the manuscript. For **Point 6**, we have referred to Table 1 in line 145, please let us know if there is another Table which we don’t refer to in the text of the manuscript. **Points 7&8**, there is no recommendation from Reviewers to cite specific previously published works, and we do not believe we have cited any papers that have been retracted, but if this is not the case then please do let us know.

On behalf of all authors, I thank you sincerely,

**Dr Daniel March**

Lecturer in Renal Science,

University of Leicester,

Room CS012 Clinical Sciences Wing,

Glenfield Hospital,

Groby Road,

Leicester,

LE3 9QP

Email: [dsm12@le.ac.uk](mailto:dsm12@le.ac.uk)

| **Editor/Reviewer** | **Comment** | **Author response/amendment** |
| --- | --- | --- |
| Reviewer 1 | It is a nice article titled - The effect of a six-month programme of intradialytic cycling on survival and hospitalisations in patients requiring hemodialysis " I find there are some grammatical errors which will need corrected but else find it acceptable | Thank you for this comment. We have proofread the manuscript, and corrected all grammatical errors. We do hope that this has improved the readability of the manuscript. |
| Reviewer 2 | This manuscript addresses an important and timely public health issue by examining the association between living alone and self-rated health among older adults in a local Japanese municipality. The focus on gender differences adds value and relevance to the research question. | We do not believe that this comment relates to our manuscript. Can the Editor please advise. |
| Reviewer 2 | The study design and statistical methods (particularly the use of logistic regression analysis stratified by gender) are appropriate and clearly reported. The discussion appropriately contextualizes the findings with previous literature, and the authors have taken care to acknowledge the limitations of the cross-sectional design. | We do not believe that this comment relates to our manuscript. Can the Editor please advise. |
| Reviewer 2 | I appreciate the clarity of the writing and the careful attention to detail throughout the manuscript. The conclusions are supported by the results, and the implications for social support and policy are well-articulated.  I have no major concerns. I recommend the manuscript for publication with no further revisions. | We do not believe that this comment relates to our manuscript. Can the Editor please advise. |
| Reviewer 3 | This is a long-term follow-study of the CYCLE-HD trial, which was a 6-month randomized controlled trial of intra-dialytic cycling. The authors examine the effect of the intervention on all-cause mortality, hospitalizations, and cardiovascular events at 5 years. There were no statistically significant effects on mortality, a composite outcome of cardiovascular mortality and hospitalizations, or other outcomes. The authors do a good job of placing their findings in the context of prior studies in a thoughtfully written discussion. They also acknowledge that CYCLE-HD, which randomized 130 participants, was underpowered for the purposes of this analysis. I have the following comments: | We would like to thank the Reviewer for this kind summary, and specific comments that we feel have significantly improved the overall quality of the manuscript. |
| Reviewer 3 | 1. It is not clear why time zero was defined differently for the intervention and control arms (lines 96-97). The time of randomization would seem to be the appropriate starting time for all participants. | Thank you for this comment, apologies that this was unclear. Cluster randomisation was in place, therefore date of randomisation for the participant is not appropriate here, as the days in which the participant dialysed were what was randomised and not the participant. Rather than use the day in which they were enrolled as the starting time, we chose the current time horizons to ensure that it included the IDC exercise programme for the IDC group and allowed direct comparison with the control group. We have clarified this in lines 99-100 (tracked changes version). Please do let us know if you would like us to provide further clarification here. |
| Reviewer 3 | 2. The text implies that hospitalizations were only captured for University Hospitals of Leicester (line 99). If so, there is the possibility of missing data due to external hospitalizations. This should be added as a limitation. | Thank you for pointing this out. Yes, that is correct we only captured hospitalisations within the University Hospitals of Leicester through the Hospital Episodes Statistics (HES) database. Resultantly, there may be missing data as a result of hospitalisations occurring outside of this National Health Service Trust. We have added this to the limitations section. Please see lines 277-279 (tracked changes version). |
| Reviewer 3 | 3. Results, lines 142-146: this text presumably refers to events occurring between the start of the study and the end of follow-up, not within the 6 months of the intervention. This should be clarified. | Withdrawal of consent was during the 6-month intervention period only. All other withdrawals reported were from the beginning of the time horizon to the end of 5-year follow up. We have clarified this in lines 146-149 (tracked changes version). |
| Reviewer 3 | 4. Line 277: Given that the effect size of reduction in hospitalizations was similar to that seen in DIATT, the lack of statistical significance may simply be due to lack of power, and does not indicate absence of clinical significance. Thus “unlikely to be beneficial” may be too strong of a statement. | Thank you for this comment. We have now changed the conclusion to highlight the exact point that the reduction in hospitalisations is similar to the DIATT trial, and the results may be due to us being underpowered. Please see lines 286-289 (tracked changes version). We would be happy to make further changes to this if you feel that this comment has not been fully addressed. |
| Reviewer 3 | 5. Table 1: both rows for mortality have incorrect values for percentages (controls, all-cause mortality 57%; IDC, CV deaths 22%). | Thank you for pointing this out. We have now corrected this, and checked the other data. |
| Reviewer 3 | 6. There are multiple typos or missing callouts throughout the manuscript (e.g. lines 139-140). | We have proof read and corrected all typos and grammatical errors. We hope that this has improved the readability of the manuscript. |
| Reviewer 3 | 7. Line 198: “physically inactive” | Thank you for this. We have now updated this. |
